# DNA methylation regulatory patterns and underlying pathways behind the co-pathogenesis of allergic rhinitis and chronic spontaneous urticaria

**DOI:** 10.3389/fimmu.2022.1053558

**Published:** 2023-01-11

**Authors:** Zijiang Yang, Puqiao Wen, Jing Chen, Jian Kang, Yaping Xiang, Shu Ding, Lihua Gao, Xiaoliang Tong, Aiyuan Guo

**Affiliations:** ^1^ Department of Dermatology, Third Xiangya Hospital, Central South University, Changsha, Hunan, China; ^2^ Xiangya School of Medicine, Central South University, Changsha, Hunan, China

**Keywords:** CD4+ T cells, DNA methylation, pollen, allergic rhinitis, chronic spontaneous urticaria

## Abstract

**Background:**

Allergic rhinitis (AR) and chronic spontaneous urticaria (CSU) are often concurrent in patients. Changes in DNA methylation affect T cell biological processes, which may explain the occurrence and progression of comorbidity. However, downstream regulatory pathways of DNA methylation in two diseases and the underlying mechanisms have not been fully elucidated.

**Methods:**

The GSE50101, GSE72541, GSE50222 and OEP002482 were mined for the identification of differentially expressed genes (DEGs) or co-expressed genes and differentially methylated genes (DMGs) in AR and CSU patients. We applied GO analysis and consensus clustering to study the potential functions and signal pathways of selected genes in two diseases. GSVA and logistic regression analysis were used to find the regulatory pathway between DNA methylation and activation patterns of CD4+ T cells. Besides, we used the Illumina 850k chip to detect DNA methylation expression profiles and recognize the differentially methylated CpG positions (DMPs) on corresponding genes. Finally, we annotated the biological process of these genes using GO and KEGG pathway analysis.

**Result:**

The AR-related DEGs were found closely related to the differentiation and activation of CD4+ T cells. The DEGs or co-expressed genes of CD4+ T cells in AR and CSU patients were also clustered using GO and KEGG analysis and we got 57 co-regulatory pathways. Furthermore, logistic regression analysis showed that the regulation of cellular component size was closely related to the activation of CD4+ T cells regulated by DNA methylation. We got self-tested data using the Illumina 850k chip and identified 98 CpGs that were differentially methylated in patients. Finally, we mapped the DMPs to 15 genes and found that they were mainly enriched in the same CD4+T cell regulating pathway.

**Conclusion:**

Our study indicated that DNA methylation affected by pollen participated in the activation patterns of CD4 + T cells, providing a novel direction for the symptomatic treatment of the co-occurrence of AR and CSU.

## 1 Introduction

AR, induced by type I hypersensitivity response upon sensitized individuals’ exposure to inhaled allergens, is regarded as one of the most common allergic disorders and affects 10% to 40% of the global population (1, 2). Characterized by sneezing, nasal congestion, and rhinorrhea, individuals suffering from moderate or severe allergic rhinitis often experience troublesome symptoms, such as learning disabilities in school-aged children and productivity impairment in adults (3–5). Several studies have shown that AR is closely related to and often co-occurs with CSU, which is defined as the spontaneous appearance of wheals, angioedema, or both for more than 6 weeks due to known or unknown causes (6, 7). It has been confirmed that the activation of CD4+T cells is the common stage in the development of AR and CSU (8–10). Through the secretion of cytokines, CD4+ T cells contribute to mast cell activation in comorbidity patients (11). Then, chemokines from mast cells lead to infiltration of inflammatory cells, which may, in turn, further activate mast cells (10, 12). The release of inflammatory mediators causes vasodilation and increases vascular permeability of nasal mucosa or skin, leading to corresponding allergic symptoms (13). Therefore, CD4+ T cells play a dominant role in the type I immune response of these two allergic diseases, the mechanism of which appears to be an interesting topic.

Studies have shown that AR and CSU have common triggers, of which pollen is the important incentive (14). In detail, data from clinical trials confirmed that there was a significant correlation between allergic reaction with pollen counts (15). According to patients’ sensitization to cyclic pollens or year-round allergens, allergic rhinitis has been classified traditionally as seasonal or perennial (11). This categorization can be useful to establish allergen specific treatment to ascertain the correct allergen desensitization (16). Moreover, as a chronic relapsing skin disease, CSU is associated with impaired skin barrier function and the atopic constitution. Various allergenic factors, such as irritants, pollen, and microbial organisms, have been found to induce the development of CSU symptoms. Besides, in the double-blind and placebo-controlled study published by Werfel et al. in 2015, it was demonstrated that exposure to pollen triggers cutaneous reactions in allergic dermatitis (17). A systematic literature review also found that positive skin-prick test (SPT) results for single or multiple aeroallergens were common in CSU patients, with multiple aeroallergen SPTs occurring at a 3.1-fold higher rate than controls (18). Although pollen was considered a key allergen in AR and CSU, the intrinsic pathogenesis of how it leads to immune responses was still unclear. Studies have shown that Bet v1, one of the most significant plant allergens in pollen, can be recognized by T cells in individuals with birch pollen allergy (19, 20). It was also widely accepted that immune responses to airborne allergens likely involve two subsets of CD4+ T cells, namely Tfh cells and Th2 cells (21). In conclusion, we will focus on the common mechanism of AR and CSU mediated by T cells under the influence of pollen.

Nowadays, the diagnosis of AR and CSU is made by medical history, physical examination and, if necessary, allergen-specific IgE testing in some patients (22). Available treatments for AR include H1- antihistamines, intranasal corticosteroids (INCS), and allergen-specific immunotherapy (AIT) (23). Except for these non−targeted therapies, there are no biomarkers available in clinical practice to predict the type (ie, phenotype or endotype), the severity of AR and the development of comorbidities (8). Besides, omalizumab was currently the only biologic therapy approved for the treatment of CSU with numerous clinical trials supporting its use in curing urticaria (24). However, nearly 40% of patients taking omalizumab for CSU continued to have moderate or poor control of symptoms, and 11.8% had no response to the drug, which warrants further research into new CSU therapies and biological targets (25, 26).

Recently, DNA methylation within the genome from allergic patients is regarded as one of the distinctive epigenetic signatures, mediating environmental effects on the development of diseases (27). Studies have found that exposure to air pollution in pregnancy correlated with newborn blood DNA methylation at over 450,000 CpG sites (CpGs), increasing the incidence of asthma (28). It is also reported that the lack of contact with bacterial antigens during one’s youth resulted in the increased occurrence of atopic dermatitis by the demethylation of the *RORC* gene (29). Therefore, DNA methylation is expected to become a new biological target of AR and CSU comorbidity, although its role in disease development has not been fully clarified. Current research suggested that it may contribute to a variety of cytological processes of T cells in different allergic diseases, including Treg deficiency, TH1 and TH2 polarization, and differentiation of CD4+ T cells (30–32). Interestingly, researchers have found that some allergic responses of AR or CSU patients can be predicted by the DNA methylation levels when exposed to grass pollen (33, 34). In conclusion, the above results indicated that pollen can affect DNA methylation in comorbidity patients, potentially regulating the biological functions of T cells.

In this study, we comprehensively analyzed genes associated with CD4+ T cells in AR-CSU patients and performed functional enrichment. Then, we used DMP analysis and GO analysis to find out the association between DNA methylation and these two types of allergic diseases. In addition, GSVA and logistic regression analysis were performed to dig out the link between the activation of CD4+ T cells and DNA methylation. Finally, we performed the detection of DNA methylation expression profiles on 10 samples using the Illumina 850k chip and functional enrichment analysis of DMGs using GO and KEGG pathway analysis. These findings shed light on the assumption that DNA methylation occurred in AR-CSU patients and regulated CD4+ T cell activation to change allergic reactions.

## 2 Materials and methods

### 2.1 DNA methylation array analysis

The peripheral blood from 24 CSU patients and 31 healthy people was collected to extract DNA. Then, Illumina 850K chip was used to detect the methylation levels of peripheral blood DNA in 6 chronic urticaria patients (3 samples were effective, 3 samples were invalid) and 4 normal controls (35). Clinical characteristics of CSU patients used for methylation assay based on microarray were shown in **Supplementary Table 1**. The array data of Illumina methylation chips (.IDAT files) were analyzed using “ChAMP” package in the R software to figure out the DMGs between patients and normal controls (36). The studies were reviewed and approved by the Ethic Committee of the Third Xiangya Hospital, Central South University. The patients/participants provided their written informed consent.

### 2.2 Data collection and processing

**Figure 1:**
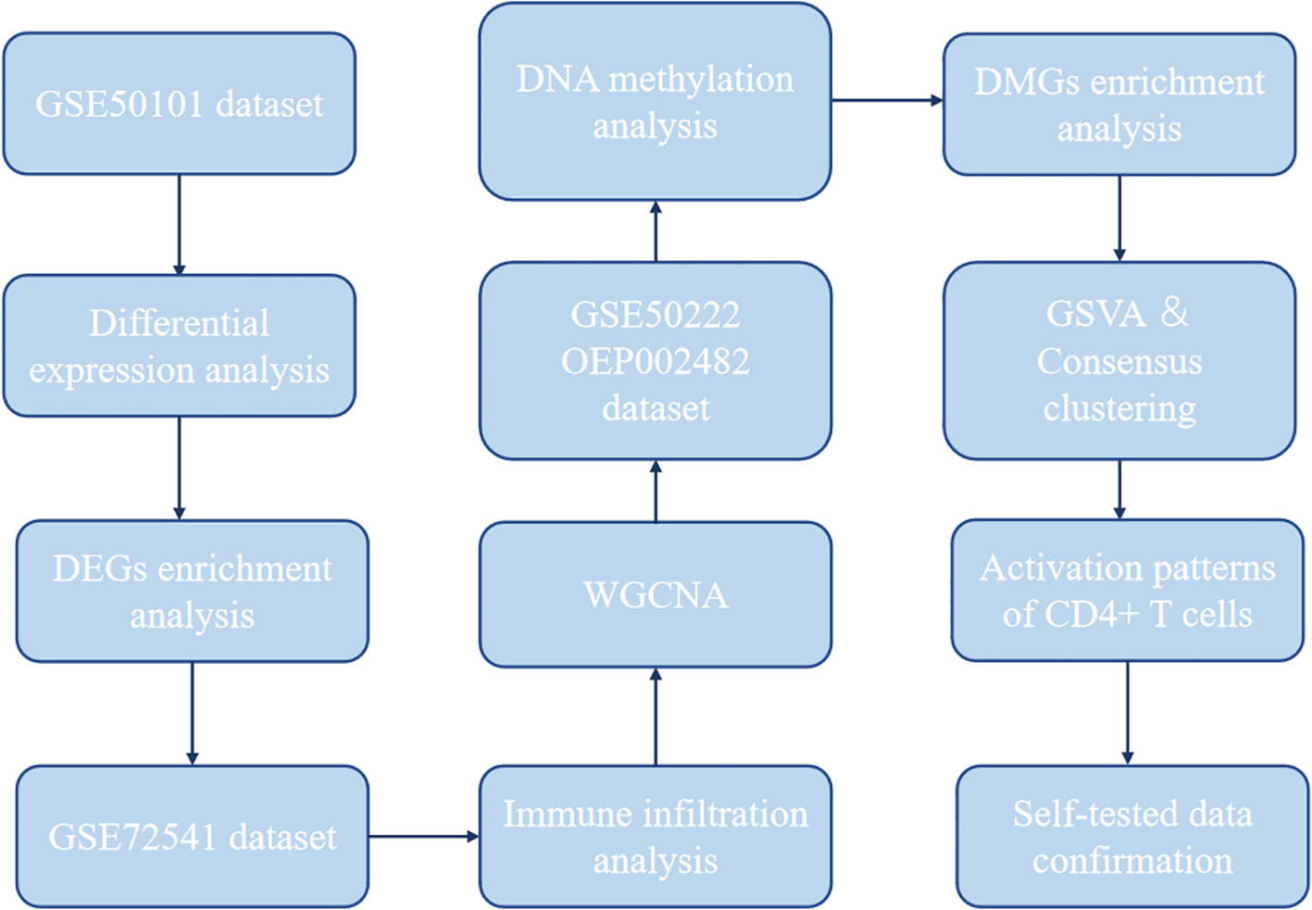
Flowchart for bioinformatics analysis of publicly available data from GEO and NGDC databases. DEGs, differentially expressed genes; WGCNA, weighted gene co-expression network analysis; DMG, differentially methylated gene; GSVA, gene set variation analysis.

Expression profiling data by array related to CD4+ T cells from AR and CSU samples were collected from GEO database (https://www.ncbi.nlm.nih.gov/geo/; GSE50101, n = 38, AR = 20, healthy control = 18; GSE72541, n = 30, CSU = 20, healthy control = 10) (37, 38). The data of genome-wide DNA methylation profile of the AR patients were available at GEO database (https://www.ncbi.nlm.nih.gov/geo/; GSE50222, n = 32, AR during pollen season = 8, AR outside pollen season = 8, healthy control =16) (37). The data of genome-wide DNA methylation profile of the CSU patients were available at National Genomics Data Center (NGDC) database (https://www.biosino.org/node; OEP002482, n = 190, CSU = 95, healthy control = 95) (34). Our workflow for bioinformatics analysis of publicly available datasets from both the GEO and NGDC databases is illustrated in **Figure 1**.

### 2.3 Differential expressed gene analysis

To determine the DEGs of AR or CSU, the R package “limma” (version 3.29.0) was used for comparison of the microarray expression profiles of cells or tissues from GEO. False discovery rate (FDR) was used to correct for multiple hypothesis testing. Genes with |log 2 (fold change) | ≥ 0 and FDR ≤ 0.05 were recognized as DEGs. The volcano plot and heatmap were depicted with the “ggplot2” package of R software.

### 2.4 Data processing for DNA methylation profiles and differentially methylated position analysis

The DNA methylation data for AR and CSU obtained from GEO and NGDC were quantified by the β value varying from 0 to 1. The probes with null values were imputed by the K-nearest neighbor (KNN) imputation procedure. Then, the methylation analysis was annotated with the required R package “IlluminaHumanMethylationEPICmanifest” on hg38 reference. The methylation β matrix was filtered and estimated and then normalized with the champ.filter function. A probe was identified as a hypermethylated probe if the difference value of probe methylation level (Δβ)between patients and normal group was greater than 0 with an adjusted p value less than 0.05, and vice versa for hypomethylated probes. Differentially methylated CpG positions (DMPs) were detected by the champ.DMP function (39). Moreover, differentially methylated genes (DMGs) were found based on the probe locations.

### 2.5 Gene function enrichment analysis

To explore the enrichment of DEGs and DMGs in potential biological processes and molecular functions from GEO, the cluster “Profiler” package (version 3.18.0) of R software was carried out, including Gene Ontology (GO) function analysis. The adjusted p < 0.05 was regarded as a statistically significant difference in the charts. The Gene Set Variation Analysis (GSVA) R package (version 1.26.0) was used to calculate normalized enrichment scores for gene sets in biological processes of CD4+ T cells associated with AR and CSU (40). These gene sets were downloaded from MSigDB database version 6.1. GO and KEGG enrichment analysis was also used to identify the pathways of DMGs in self-tested samples.

### 2.6 Immune infiltration analysis

Single-sample Gene Set Enrichment Analysis (ssGSEA) (www.gsea-msigdb.org/gsea/index.jsp) was carried out to calculate the immune infiltration and evaluate the proportion of 24 infiltrating T lymphocytes in each CSU sample according to their respective markers. These markers were searched from CellMarker database (http://biocc.hrbmu.edu.cn/CellMarker/) and the detailed information was shown in **Supplementary Table 3** (41). Correlations between different cell subsets and clinical characteristics were evaluated using Spearman’s rho and presented with a p-value (42).

### 2.7 Weighted gene co-expression network analysis

WGCNA can be used to find clusters or modules of highly correlated genes and link modules to one another and to external sample traits (43). Co-expression networks of CSU were built using WGCNA in R. To construct the networks, the adjacency matrix was converted into the topological overlap matrix (TOM) when the power of β was equal to 15 (R^2^ = 0.9) so that the final matrix followed an approximate scale-free topology. The WGCNA dynamic tree-cut algorithm was used to detect network modules. In order to determine which modules and corresponding processes were most associated with CD4+ T cells in CSU patients, we ran singular value decomposition on each module’s expression matrix and used the resulting module eigengene (ME), which is equivalent to the first principal component, to represent the overall expression profiles for each module. Similar modules were merged following a height cutoff of 0.25.

### 2.8 Statistical analysis

The statistical analyses were conducted using R software (version 4.1.2; https://www.r-project.org/). The data was normalized by “normalizeBetweenArrays” limma package to acquire the expression of DEGs. The correlation analysis was performed by using the Spearman method. Consensus clustering was performed to determine the best clustering number of the chronic CD4+ T cell activation patterns (44). The regression analysis was used to confirm the relationship between methylation regulatory pathways and CD4+ T cell activation patterns and p < 0.05 was considered statistically significant. “ggplot2” package of R software was used for data visualization.

## 3 Result

### 3.1 Identification and functional enrichment analysis of DEGs in AR-related CD4+T cells

**Figure 2:**
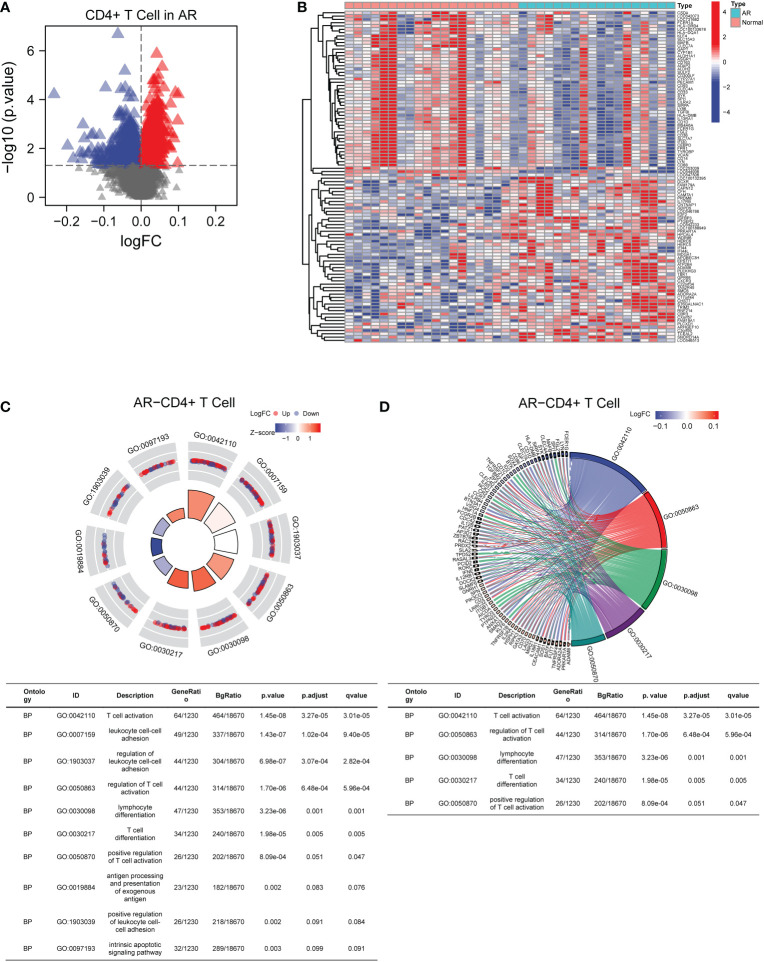
Identification and functional enrichment analysis of DEGs in AR-related CD4+T cells. **(A)** The volcano plot screened DEGs in CD4+T cells from AR patients. **(B)** The heat map displayed the top 100 DEGs with correlation. **(C)** GO pathway enrichment analysis data for DEGs (the top 10 were shown in the circle plot). **(D)** GO chord plot of DEGs (the top 5 were shown). AR, allergic rhinitis; GO, Gene Ontology.

In the GSE50101 dataset, we screened for DEGs in CD4+ T cells to assess the expression levels of each gene in AR patients and normal people (**Figure 2A**). The full description of DEGs was depicted in **Supplementary Table 2**. In addition, the top 100 genes with correlation were shown in the heat map (**Figure 2B**). The results showed that there were significant genetic differences in CD4+ T cells between AR patients and normal controls. GO pathway enrichment analysis was performed to explore the potential biological functions of the above DEGs (**Figure 2C, D**). A total of 169 GO terms of biological processes, 12 GO terms of cellular component, and 5 GO terms of molecular function were identified. Among these, the top 10 GO terms were exhibited in the circle plot, where we found the DEGs were closely related to biological processes including T cell activation and differentiation. The p-value for expression of the DEGs in AR versus normal controls was statistically significant in the GSE50101 dataset (p-value < 0.05).

### 3.2 Overview of the distribution of immune cell subtypes in CSU patients

**Figure 3:**
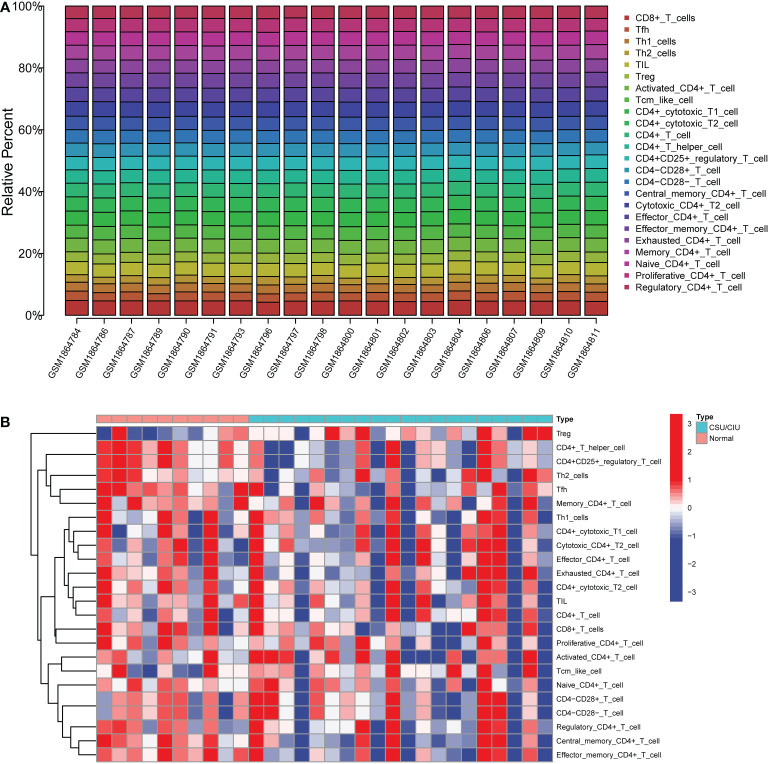
The profiles of immune cell subtype distribution and correlation in GSE72541 cohorts. **(A)** Stacked graph of different types of immune cell proportion in CSU patients. **(B)** Heatmap of immune cell distribution in 30 samples. CSU, chronic spontaneous urticaria.

In order to figure out the proportion of various types of immune cells in CSU patients, the stacked graph was depicted to show the immune cell subtype distribution. It was observed that activated CD4+T cells, CD4+ cytotoxic cells, CD4+T cells and CD4+T helper cells were the main infiltrating cells (**Figure 3A**). To have an overview of CSU patients’ immunity, we used the heatmap to show the differences in immune cell numbers between the CSU/CIU group and the normal control group (**Figure 3B**). The result indicated that there was a general decrease in immune infiltration in the CSU/CIU group, suggesting a weakened immune system mainly associated with CD4+ T in the patients. The above results suggested that CD4+ T cells played a major role in the immune infiltrating microenvironment in CSU patients and were closely related to the occurrence and development of this disease.

### 3.3 Establishment of scale-free network of CSU-related DEGs and identification of main module associated with immune-infiltration

**Figure 4:**
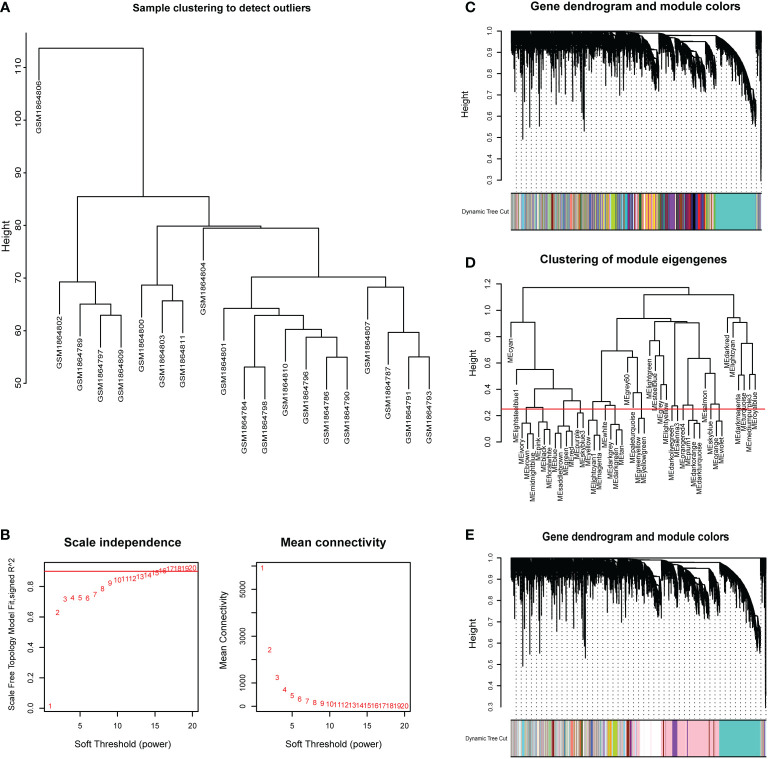
Sample dendrogram and clustering dendrogram of WGCNA. **(A)** Cluster samples to detect outliers. **(B)** Plot scale-free topology to determine soft threshold (power). **(C)** The identified co-expression modules and the dendrogram. **(D)** Set the criteria to merge similar modules. **(E)**The dynamic cut tree after merging modules.

**Figure 5:**
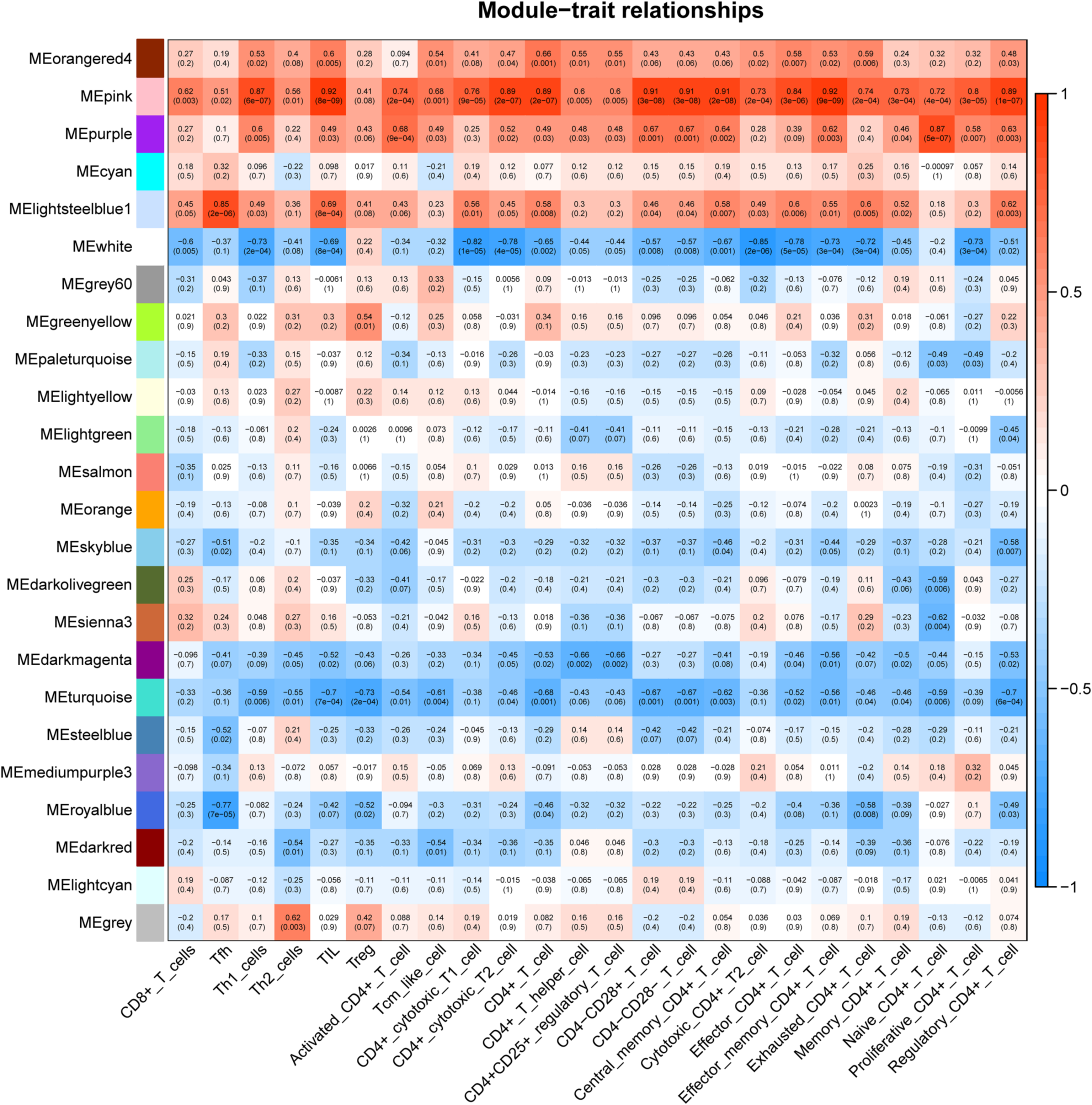
Correlation between diverse subtypes of immune-infiltrating T cells and different gene modules.

CSU samples (n=20) were clustered to detect the outliers while we did not remove any objects owing to the small sample size (**Figure 4A**). To build a scale-free network of genes in CSU patients, β=15 (scale-free R^2>0.90) was set as the most appropriate soft threshold (power). (**Figure 4B**). Furthermore, the dynamic hybrid cutting method was performed to construct a hierarchical clustering tree, where similarly expressed genes were collected to form a gene module (**Figure 4C**). Then we selected a height of 0.25 with a correlation greater than 0.75 to integrate modules with similar expression patterns. (**Figure 4D**). A total of 45 modules were generated after merging. (**Figure 4E**). Based on the T cell markers found in CellMarker database, we regarded the genes for a series of markers of the same type of T cell as a gene set. The markers for each kind of T cell were shown in **Supplementary Table 3**. Then, the proportion of immune cells in each sample was calculated by ssGSEA and 24 common T cell subtypes were included. The proportion of each T cell subtype in CSU patients was extracted as phenotypic data and its association with the WGCNA modules were analyzed (**Figure 5**). The high correlations were mostly found between genes in the pink module and urticaria-related immune-infiltrating cells (R^2≧0.90, p < 0.01). A total of 6735 genes in the pink module were shown in the **Supplementary Table 4**. According to **Figure 3**, we clarified the leading role and high correlation of CD4+ T cells in the immune infiltration of CSU. Then, through WGCNA in **Figure 4** and **Figure 5**, we found genes in the pink module had a high correlation with immune infiltration of CD4+ T cells in CSU patients. Hence, we speculated that genes in the pink module may potentially influence the level of immune infiltration of CD4+ T cells, which in turn regulated disease development. They would be used for gene enrichment analysis.

### 3.4 Gene set enrichment analysis of AR and CSU related DMGs in pollen season

**Figure 6:**
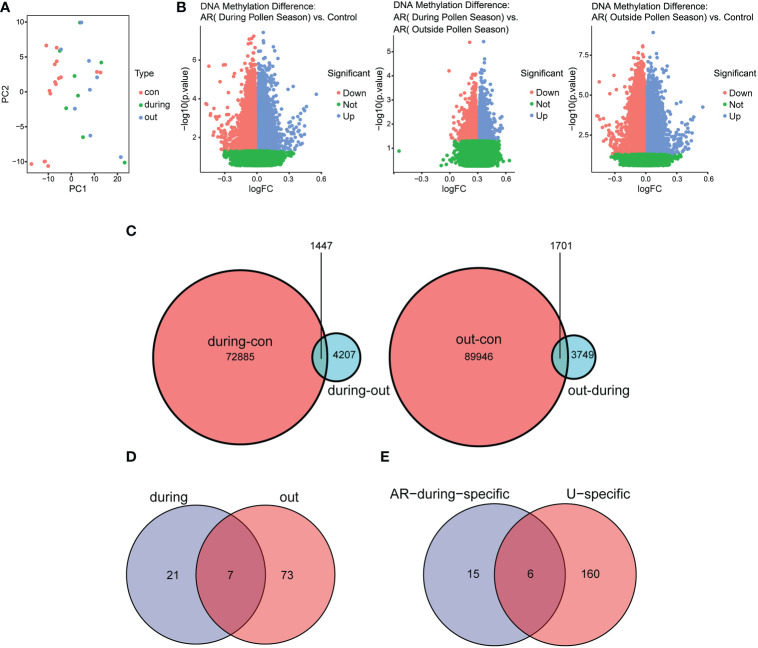
Identification of DMPs in CD4+ T cells from AR and CSU patients. **(A)** The PCA plot of patients with AR during or outside pollen season and normal control samples. **(B)** The volcano plot of pollen season related DMPs in AR patients and normal control samples. **(C)** The Venn diagram showed pollen-related and pollen-independent DMPs. **(D)** The Venn diagram showed the number of regulatory pathways of AR genes during or outside the pollen season. **(E)** The Venn diagram showed the number of common signaling pathways regulating AR and CSU during the pollen season. DMPs, differentially methylated CpG positions.

**Figure 7:**
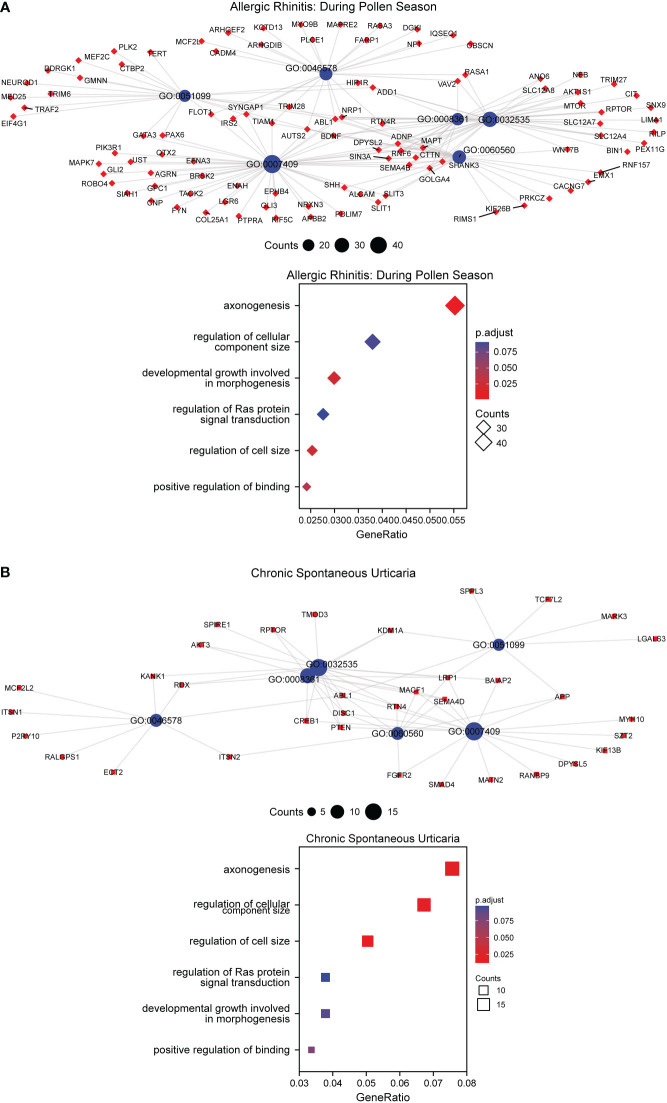
GO enrichment analysis of DMGs in AR and CSU patients. **(A)** GO enrichment analysis of DMGs and bubble chart of regulating pathways in AR patients. **(B)** GO enrichment analysis of DMGs and bubble chart of regulating pathways in CSU patients.

To explore whether the gene expression profiles in AR correlated with pollen, principal component analysis (PCA) was performed to separate the samples from GSE50222 (**Figure 6A**). The result showed that the dataset supported the classification of samples into three categories, including the control group, during the pollen season group and outside the pollen season group. To find the pollen season related DMGs, we analyzed the DMGs from AR patients during or outside pollen season and healthy individuals and displayed them on the volcano plot (**Figure 6B**). The results indicated a total of 74,332 DMPs between AR during pollen season and Control (during-con), 11,104 between AR during pollen season and AR outside pollen season (during-out: hypermethylated DMPs during pollen season compared to outside pollen season and out-during: hypomethylated DMPs during pollen season compared to outside pollen season), and 91,647 between AR outside pollen season and Control (out-con). As shown in the Venn diagram, we found 1447 pollen-related DMPs and 1701 pollen-independent DMPs (**Figure 6C**). Further, GO and KEGG analysis was performed to explore the potential biological functions of DMGs in AR and CSU based on the GSE50222 and OEP002482. The Venn diagram analysis of the pathways showed 21 unique pathways for season-specific AR (ssAR) and 6 shared pathways between ssAR and season-specific CSU (ssCSU) (**Figures 6D, E**). Then, the shared pathways were determined and visualized using the network of GO to prohibit the specific genes associated with ssAR or ssCSU. GO analysis revealed that the genes were mainly enriched in the axonogenesis signaling pathway and the regulation of cellular component size signaling pathway (**Figures 7**). The complete results of the GO analysis and corresponding genes could be found in **Supplementary Table 5**-**7**.

### 3.5 Categorization of AR and CSU Co-regulated pathways and corresponding patterns of chronic CD4+ T cell activation

**Figure 8:**
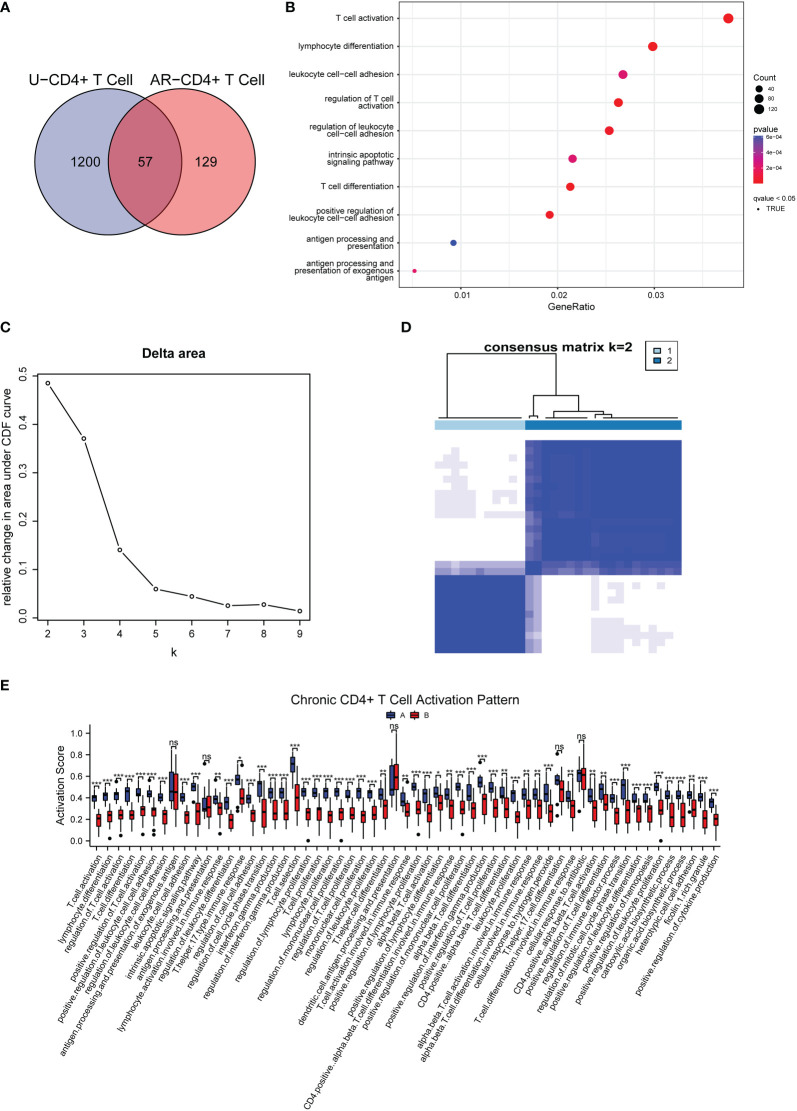
Categorization of AR and CSU co-regulated pathways and corresponding patterns of chronic CD4+ T cell activation. **(A)** Venn diagram exhibited the shared signaling pathways associated with CD4+ T cells in AR and CSU patients. **(B)** GSVA shown by bubble plots. **(C)** Relative change in area under the CDF curve under different values of (k) **(D)** The consensus matrix for k = 2. **(E)** Box plot showing differences of two types of chronic CD4+ T cell activation patterns.

Through GO and KEGG analysis, we found 57 common regulatory pathways in CD4+ T cells from AR and CSU patients (**Figure 8A**). The complete results of the GO and KEGG analysis could be found in **Supplementary Table 8**. As depicted in the bubble plot after GSVA, 120 or more genes were involved in the process of T cell activation and lymphocyte differentiation, respectively; 80 or more genes were associated with T cell activation, regulation of cell adhesion, T cell differentiation, and antigen processing, respectively; 40 or more genes were involved in antigen processing and presentation (**Figure 8B**). The genes in CD4+T cells related to AR and CSU were collected for consensus clustering, whose process was shown in **Figures 8C, D**. As shown in **Figures 8C, D**, in accordance with the approach for consensus clustering, when k = 2, the co-regulated pathway cohort could be separated into two subgroups which were different and did not overlap. The 57 signaling pathways of each sample were scored using GSVA (**Figure 8B**), and the pathways were divided into 2 groups according to scores. The boxplot results showed that the activation level of group A was higher than that of group B (**Figure 8E**). Thus, patients with allergic comorbidities had chronic CD4+ T cell-specific activation patterns regulating disease progression. By establishing a logistic regression model of 30 comorbidity patient samples, the relationship between 57 regulatory pathways related to T cell activation and 6 differentially methylated gene enrichment pathways was found (**Supplementary Table 9**). Then, according to the OR value, confidence and P value, the cellular component size regulation pathway was finally obtained, which not only was regulated by the methylated DNA but also changed the T cell activation patterns.

### 3.6 Identification and verification of differentially methylated CpG positions in 10 self-test samples

**Figure 9:**
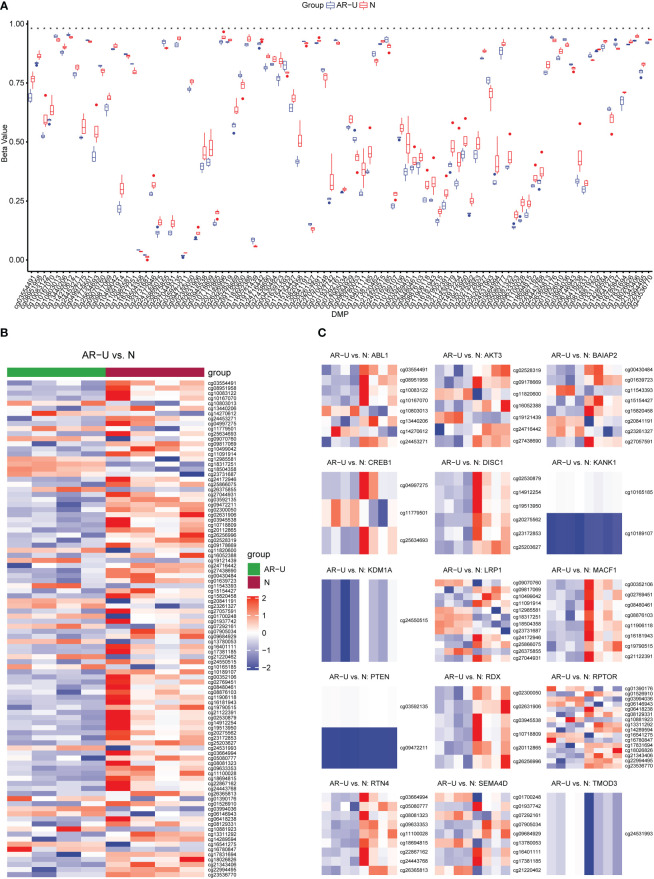
Identification and verification of DMPs. **(A)** Overview of methylation rates for 98 CpGs in the box plot. **(B)** Heatmap of 98 differentially methylated CpGs between AR-CSU patients and healthy individuals. **(C)** Heatmap of DNA methylation levels of different CpGs on the corresponding genes.

**Figure 10:**
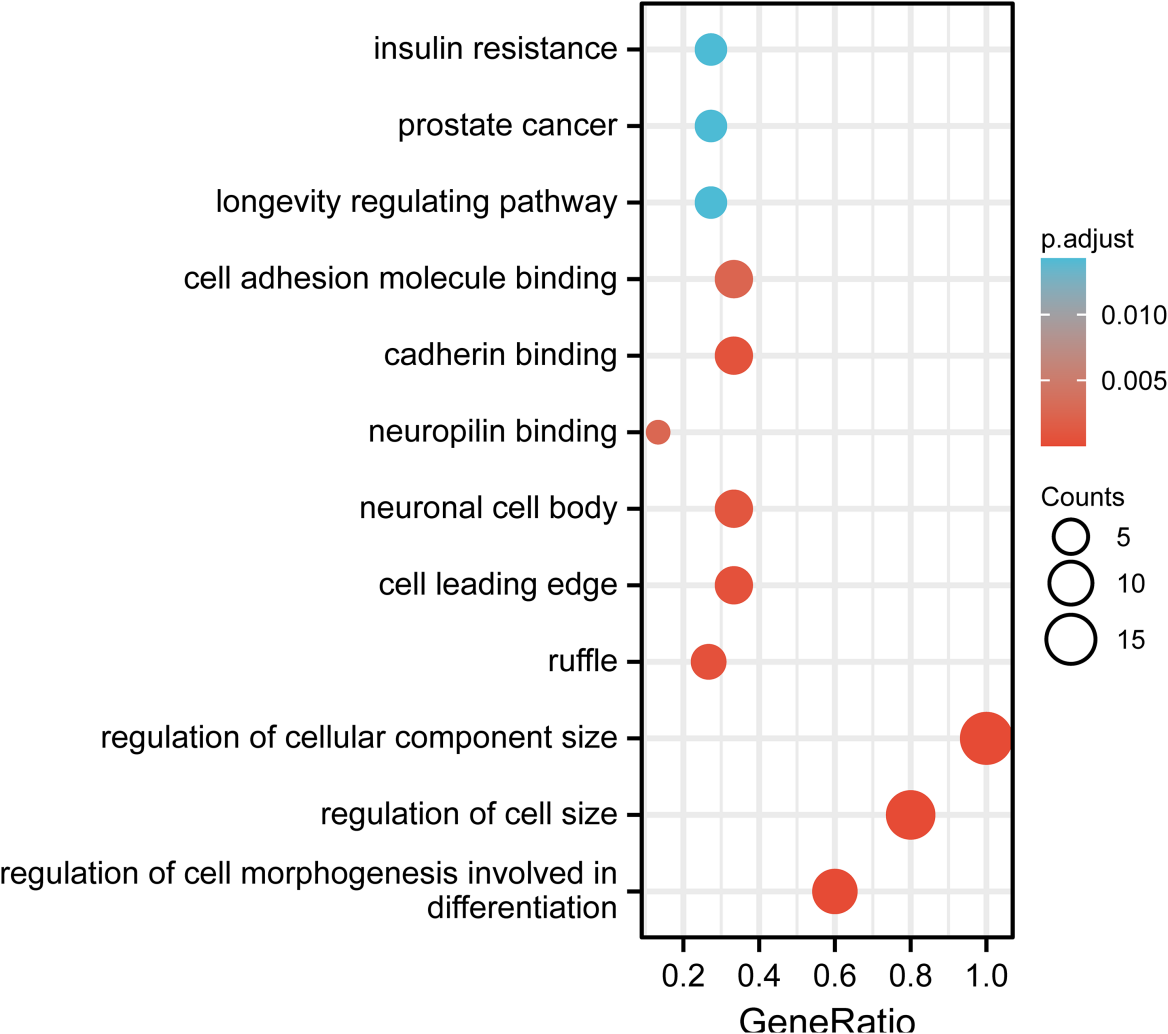
GO terms and KEGG pathways enrichment analysis of 15 DMGs in self-tested samples.

In this study, we performed the detection of DNA methylation expression profiles on 10 samples (AR-CSU: n = 6; normal: n = 4) using the Illumina 850k chip. As shown in the boxplot, we examined the methylation rates for all 98 CpG positions and found differences between AR-CSU patients and healthy individuals were distinguishable at most CpG positions (**Figure 9A**). Then, we used the heatmap to identify DMPs and marked them on the corresponding genes (**Figure 9B, C**). In conclusion, we recognized 26 hyper-methylated positions and 72 hypo-methylated positions among 98 DMPs and determined 15 DMGs based on probe location in corresponding genes. Finally, we conducted GO terms and KEGG pathways analysis of these genes to identify their biological functions (**Figure 10**). The analysis suggested that 15 DMGs were mainly involved in the regulation of cellular component size, the regulation of cell size and the regulation of cell morphogenesis involved in differentiation, which were in good agreement with the database analysis results and finally confirmed the assumption that DNA methylation occurred in AR-CSU patients and regulated CD4+ T cells activation to change allergic reactions.

## 4 Discussion

AR and CSU often co-occur in the same individual, which can be partly explained by the Th2 immune response (45–47). It has been reported that pollen was regarded as one of the most common causes of these two diseases, leading to the exacerbation of symptoms (48, 49). Besides, in pollen season, DNA methylation, a kind of epigenetic modification, can be affected by environmental aeroallergens (50). Recently, it has been recognized as one of the most distinctive epigenetic signatures in allergic diseases such as allergic rhinitis and atopic dermatitis (51, 52). In allergic disease patients, DNA methylation can change cytological functions such as differentiation and activation of T cells and regulate the process of disease development (53). However, the specific pathways that DNA methylation regulates comorbidity and the potential methylation sites associated with allergic diseases have not yet been discovered. In this study, we systematically analyzed DEGs in the CD4+ T cells associated with AR and CSU in the GSE50101 and GSE72541. WGCNA was applied to identify immune infiltration in CSU. Moreover, GSVA and consensus clustering were performed. Finally, Illumina 850k chip was used to detect DNA methylation expression profiles on 10 samples. These findings clarified the essential regulatory pattern of DNA methylation under the influence of environmental factors in comorbidity.

Several researchers reported the essential contribution of CD4+ T cells in allergic diseases (54). Through producing and secreting type 2 cytokines such as IL-4, IL-5 and IL-13, Th2 cells activate B cells to class-switch to IgE and incentive mast cells to release inflammatory mediators, leading to the occurrence of allergic diseases (47, 55). Hence, in the following study, we identified the DEGs in CD4+ T cells between AR patients and healthy individuals. Then, GO analysis was performed, and the results showed that AR-related DEGs were closely associated with cytological functions such as T cell activation and differentiation, which contribute to the regulation of immune metabolism and release of cytokines in allergic diseases (56). Then we assessed cell subtypes in the immune-infiltrating microenvironment from CSU samples. As shown in **Figure 3**, we found that, in spite of the decline in the immune function of CSU patients, CD4+ T cells were highly enriched within the immune cell subtypes, which indicated the critical role of CD4+ T cells in the development of CSU disease. Then, WGCNA was performed on the related genes in the CSU patients and 45 modules were identified, of which the pink module was highly associated with the infiltrating immune cells, mainly including various subsets of CD4+ T cells. Thus, the genes in the pink module were likely to mediate the development of CSU by affecting different subsets of CD4+ T cells. The above results elucidated that the co-occurrence of AR and CSU was closely related to CD4+ T cells.

Pollen is considered to be one of the most common allergens in AR (57). As is reported, seasonal climate change increases the length and intensity of the pollen season, which have a significant impact on the millions of AR patients (58). Nowadays, AR can be classified as a seasonal disease, according to the rapid and reproducible onset and offset of symptoms in association with pollen exposure (59). In our study, PCA was performed to divide the AR samples and healthy controls into three categories including the control group, during the pollen season and outside the pollen season. Through the mutual comparison between categories, we found that a total of 1447 season-specific DMPs were differentially methylated using DMP analysis. Studies have shown that the specific exposures to aeroallergen can prime the changes in DNA methylation which led to the significant differences between AR patients and healthy controls in epigenetics (60). North et al. found that DNA methylation patterns could predict the severity of symptoms after exposure to grass allergens, of which DNA methylation of the *SLFN12* was significantly associated with AR (33). Zhang et al. also assessed genome-wide DNA methylation patterns and allergic sensitization among adolescents and found that methylation at cg10159529 was strongly correlated with allergic diseases (61). All these findings indicated that DNA methylation regulation may be the underlying etiology of pollen-induced seasonal AR. Besides, GO analysis was performed to explore the potential biological functions of selected DMGs. Venn showed 6 common regulatory pathways and the corresponding genes were exhibited after GO analysis. The result from **Figure 7** depicted that DMGs from AR and CSU patients were enriched in pathways related to regulation of cellular component size. In order to further explore whether the pathway is activated or deactivated after DNA methylation, we measured the methylation level of the genes in 6 common regulatory pathways. According to **Supplementary Table 7**, RDX, AKT3, RPTOR were hypomethylated, while LRP1, SEMA4D were hypermethylated. Previous studies have reported that the methylation levels of genes were generally inversely correlated with gene expression (62). Therefore, we inferred that regulation of cellular component size was up-regulated in AR and CSU patients. The above results suggested that the occurrence of AR and CSU was related to DNA methylation differences, and the common regulatory pathway under epigenetic factors was the potential cause of pollen-induced comorbidity.

Studies have shown that DNA methylation can affect the proliferation, activation, and differentiation of T cells (63, 64). It has also been confirmed that the control of DNA methylation during CD4 T cell differentiation and function was significant in human cells (65). In immune infiltration shown in **Figure 3**, we have found different subtypes of CD4+ T cells, containing Th1, Th2, Treg, naïve T cells and so on. Th1 cells are formed by differentiation of naïve T cells, which is mediated by methylation of the IL-4 gene promoter. Th2 cells have been shown to reactivate the synthesis of IFN-γ *via* demethylation of specific gene sites (66). Besides, Polyxeni Ntontis et al. found that the activation of the regulatory T cells’ suppressive role required demethylation of the FOXP3 gene (67). Therefore, we speculated that the abnormal biological function of T cells caused by DNA methylation was the pathogenesis of allergic diseases. In this study, we performed GO functional enrichment on specific genes in AR and CSU-related CD4+T cells. Through the Venn diagram intersection, a total of 57 common regulatory pathways were found. We then performed GSVA and consensus clustering of these pathways and finally identified two groups, A and B. It was surprising to find that the patients in group A were consistently expressed at higher levels in immune infiltrating cells than group B, thus establishing a pattern of activation of CD4+ T cells regulating disease progression in patients with allergic comorbidities. To further investigate the specific molecular mechanism by which DNA methylation regulates CD4+T cells, logistic regression analysis was applied to establish a correlation between 57 pathways and the above 6 common regulatory pathways. The analysis suggested that regulation of cellular component size was the critical signal pathway between DNA methylation and the activation of CD4+ T cells.

Finally, the result showed that there were 98 DMPs shared by AR and CSU occurring in the pollen season. The heatmap in **Figure 9B** depicted that patients generally had lower methylation levels with 26 hyper-methylated positions and 72 hypo-methylated positions. We found that 15 genes were differentially methylated according to probe location and they were mainly enriched in the regulation of cellular component size. The significant differences of methylation confirmed its potential role in regulating the development of this comorbidity.

Nowadays, AIT therapy was still the mainstream clinical treatment for AR and CSU diseases (68). It remained an important goal to predict the efficacy of AIT and find additional immune biomarkers to provide individualized treatment for AIT (69). In our study, we first explored the co-pathogenesis of AR and CSU during the pollen season. Induced by pollen, the level of DNA methylation in patients decreased, which altered the activation of CD4+ T cells in the immune-infiltrating microenvironment and exacerbated the disease condition. Thus, it was expected to develop DMPs in comorbidity as novel biomarkers, which are critical for selecting the proper treatment for patients and enabling precision medicine (70).

However, there were still some inevitable limitations in our study. Even though we have identified DEGs and DMGs using bioinformatics analysis to elucidate the common pathogenesis of AR and CSU, more *in vivo* experiments are needed as direct cytological evidence to demonstrate the exact role of DNA methylation on T cells. In addition, after DNA methylation, further studies were needed to elucidate the activation or deactivation of downstream pathways and their impact on the biological processes of T cells. In the following research, we plan to up or down regulated the methylation levels at different CpG sites and explore their variation in proliferation, activation, and differentiation of T cells *in vitro* and vivo, attempting to find out possible treatments for pollen-induced seasonal AR and CSU at the epigenetic level.

## 5 Conclusion

In AR and CSU patients, DEGs were closely related to pathways that regulate the chronic activation of CD4+T cells. We then found the association between DNA methylation and two allergic diseases. Furthermore, we analyzed the relationship between T cells and DNA methylation and found that the regulation of cellular component size played a bridging role between them. Self-tested data was collected using the Illumina 850k chip and we identified 98 DMPs in patients. Finally, we mapped the DMPs to 15 genes and found that they were mainly enriched in the above CD4+T cell regulating pathway.

## Data availability statement

The datasets presented in this study can be found in online repositories. The names of the repository/repositories and accession number(s) can be found in the article/**Supplementary Material**.

## Ethics statement

The studies involving human participants were reviewed and approved by the ethics committee of the Third Xiangya Hospital, Central South University. The patients/participants provided their written informed consent to participate in this study.

## Author contributions

ZY, PW, JC performed the literature search, reviewed articles and completed the data analysis. JK and YX wrote the manuscript. SD and LG reviewed the articles and provided secondary reviews during the manuscript preparation. XT, and AG contributed to picture integration. All authors have read and approved this information before submission.
