# Supplementary material for: DNA methylation regulatory patterns and underlying pathways behind the co-pathogenesis of allergic rhinitis and chronic spontaneous urticaria
Source: Front Immunol. 2023 Jan 11;13:1053558. doi: 10.3389/fimmu.2022.1053558 (PMC9875140; doi:10.3389/fimmu.2022.1053558)
Supplement: Supplementary file 1 [file DataSheet_1.zip › Supplementary Table 5, 6 and 8.docx]

Supplementary Material

# Supplementary Tables

**Supplementary Table 1.** Clinical characteristics of CSU patients used for methylation assay based on microarray (For a full description of the statistics see additional Excel file **Supplementary Table 1**).

**Supplementary Table 2.** Differential expression analysis of genes in CD4+ T cells of AR patients (For a full description of the statistics see additional Excel file **Supplementary Table 2**).

**Supplementary Table 3.** Markers for 24 types of T cells in CSU patients (For a full description of the statistics see additional Excel file **Supplementary Table 3**).

**Supplementary Table 4.** 6735 genes in the pink module in WGCNA (For a full description of the statistics see additional Excel file **Supplementary Table 4**).

| **Ontology** | **ID** | **Description** | **GeneRatio** | **BgRatio** | **p.value** | **p.adjust** | **qvalue** |
| --- | --- | --- | --- | --- | --- | --- | --- |
| BP | GO:0007409 | axonogenesis | 48/869 | 468/18670 | 2.54e-07 | 0.001 | 0.001 |
| BP | GO:0008361 | regulation of cell size | 22/869 | 179/18670 | 3.12e-05 | 0.025 | 0.024 |
| BP | GO:0060560 | developmental growth involved in morphogenesis | 26/869 | 235/18670 | 3.97e-05 | 0.025 | 0.024 |
| BP | GO:0051099 | positive regulation of binding | 21/869 | 179/18670 | 9.27e-05 | 0.042 | 0.039 |
| BP | GO:0032535 | regulation of cellular component size | 33/869 | 370/18670 | 2.80e-04 | 0.088 | 0.083 |
| BP | GO:0046578 | regulation of Ras protein signal transduction | 24/869 | 238/18670 | 3.21e-04 | 0.091 | 0.086 |

**Supplementary Table 5.** Visualization results of GO functional enrichment analysis of specific genes associated with AR.

| **Ontology** | **ID** | **Description** | **GeneRatio** | **BgRatio** | **p.value** | **p.adjust** | **qvalue** |
| --- | --- | --- | --- | --- | --- | --- | --- |
| BP | GO:0008361 | regulation of cell size | 12/238 | 179/18670 | 3.32e-06 | 0.012 | 0.011 |
| BP | GO:0032535 | regulation of cellular component size | 16/238 | 370/18670 | 2.29e-05 | 0.017 | 0.015 |
| BP | GO:0007409 | axonogenesis | 18/238 | 468/18670 | 3.36e-05 | 0.021 | 0.018 |
| BP | GO:0051099 | positive regulation of binding | 8/238 | 179/18670 | 0.002 | 0.078 | 0.068 |
| BP | GO:0060560 | developmental growth involved in morphogenesis | 9/238 | 235/18670 | 0.003 | 0.091 | 0.080 |
| BP | GO:0046578 | regulation of Ras protein signal transduction | 9/238 | 238/18670 | 0.004 | 0.097 | 0.085 |

**Supplementary Table 6.** Visualization results of GO functional enrichment analysis of specific genes associated with CSU.

**Supplementary Table 7.** Differentially methylated genes and corresponding methylated sites in AR and CSU patients (For a full description of the statistics see additional Excel file **Supplementary Table 7**).

| ONTOLOGY | | ID | | Description | GeneRatio | | | BgRatio | p.value | p.adjust | | qvalue | | Count |
| --- | --- | --- | --- | --- | --- | --- | --- | --- | --- | --- | --- | --- | --- | --- |
| BP | GO:0042110 | | T cell activation | | | 159/4223 | 487/18723 | | 1.44E-07 | 0.0000214 | 0.0000195 | | 159 | |
| BP | GO:0030098 | | lymphocyte differentiation | | | 126/4223 | 374/18723 | | 4.37E-07 | 0.0000486 | 0.0000444 | | 126 | |
| BP | GO:0050863 | | regulation of T cell activation | | | 111/4223 | 329/18723 | | 0.00000192 | 0.00016589 | 0.0001515 | | 111 | |
| BP | GO:0030217 | | T cell differentiation | | | 90/4223 | 257/18723 | | 0.00000318 | 0.00023001 | 0.00021005 | | 90 | |
| BP | GO:0050870 | | positive regulation of T cell activation | | | 76/4223 | 216/18723 | | 0.0000148 | 0.00085173 | 0.00077783 | | 76 | |
| BP | GO:1903039 | | positive regulation of leukocyte cell-cell adhesion | | | 81/4223 | 239/18723 | | 0.000037 | 0.00174516 | 0.00159375 | | 81 | |
| BP | GO:1903037 | | regulation of leukocyte cell-cell adhesion | | | 107/4223 | 336/18723 | | 0.0000485 | 0.00211413 | 0.00193071 | | 107 | |
| BP | GO:0019884 | | antigen processing and presentation of exogenous antigen | | | 22/4223 | 47/18723 | | 0.0002079 | 0.00707777 | 0.00646369 | | 22 | |
| BP | GO:0007159 | | leukocyte cell-cell adhesion | | | 113/4223 | 371/18723 | | 0.00022998 | 0.00774468 | 0.00707273 | | 113 | |
| BP | GO:0097193 | | intrinsic apoptotic signaling pathway | | | 91/4223 | 288/18723 | | 0.00023127 | 0.00774637 | 0.00707428 | | 91 | |
| BP | GO:0019882 | | antigen processing and presentation | | | 39/4223 | 106/18723 | | 0.00061545 | 0.01734955 | 0.01584427 | | 39 | |
| BP | GO:0002285 | | lymphocyte activation involved in immune response | | | 63/4223 | 194/18723 | | 0.00091453 | 0.02392749 | 0.0218515 | | 63 | |
| BP | GO:1902105 | | regulation of leukocyte differentiation | | | 84/4223 | 279/18723 | | 0.00196672 | 0.04239685 | 0.03871842 | | 84 | |
| BP | GO:0072538 | | T-helper 17 type immune response | | | 17/4223 | 38/18723 | | 0.00199271 | 0.04266189 | 0.03896047 | | 17 | |
| BP | GO:0022407 | | regulation of cell-cell adhesion | | | 127/4223 | 448/18723 | | 0.00221037 | 0.04668003 | 0.04262999 | | 127 | |
| BP | GO:1901987 | | regulation of cell cycle phase transition | | | 111/4223 | 390/18723 | | 0.00350225 | 0.06631916 | 0.06056518 | | 111 | |
| BP | GO:0032609 | | interferon-gamma production | | | 38/4223 | 112/18723 | | 0.00383941 | 0.07183034 | 0.06559821 | | 38 | |
| BP | GO:0032649 | | regulation of interferon-gamma production | | | 38/4223 | 112/18723 | | 0.00383941 | 0.07183034 | 0.06559821 | | 38 | |
| BP | GO:0045058 | | T cell selection | | | 20/4223 | 50/18723 | | 0.00419571 | 0.0748977 | 0.06839944 | | 20 | |
| BP | GO:0050670 | | regulation of lymphocyte proliferation | | | 68/4223 | 225/18723 | | 0.00449768 | 0.07783476 | 0.07108167 | | 68 | |
| BP | GO:0042098 | | T cell proliferation | | | 61/4223 | 199/18723 | | 0.00487985 | 0.08375059 | 0.07648423 | | 61 | |
| BP | GO:0046651 | | lymphocyte proliferation | | | 84/4223 | 288/18723 | | 0.00509285 | 0.08621869 | 0.07873819 | | 84 | |
| BP | GO:0032944 | | regulation of mononuclear cell proliferation | | | 68/4223 | 227/18723 | | 0.00564111 | 0.09297383 | 0.08490725 | | 68 | |
| BP | GO:0042129 | | regulation of T cell proliferation | | | 53/4223 | 171/18723 | | 0.00650047 | 0.10175354 | 0.09292522 | | 53 | |
| BP | GO:0032943 | | mononuclear cell proliferation | | | 84/4223 | 291/18723 | | 0.00682078 | 0.10463495 | 0.09555663 | | 84 | |
| BP | GO:0070663 | | regulation of leukocyte proliferation | | | 72/4223 | 245/18723 | | 0.00740997 | 0.11123885 | 0.10158756 | | 72 | |
| BP | GO:0042093 | | T-helper cell differentiation | | | 24/4223 | 66/18723 | | 0.00755394 | 0.11258622 | 0.10281803 | | 24 | |
| BP | GO:0002468 | | dendritic cell antigen processing and presentation | | | 8/4223 | 15/18723 | | 0.00913413 | 0.12673853 | 0.11574246 | | 8 | |
| BP | GO:0002286 | | T cell activation involved in immune response | | | 37/4223 | 114/18723 | | 0.00954123 | 0.13121829 | 0.11983354 | | 37 | |
| BP | GO:0050671 | | positive regulation of lymphocyte proliferation | | | 43/4223 | 137/18723 | | 0.01049442 | 0.14000053 | 0.12785383 | | 43 | |
| BP | GO:0046631 | | alpha-beta T cell activation | | | 48/4223 | 156/18723 | | 0.01067471 | 0.14149673 | 0.12922022 | | 48 | |
| BP | GO:0045621 | | positive regulation of lymphocyte differentiation | | | 34/4223 | 104/18723 | | 0.01119646 | 0.14685045 | 0.13410944 | | 34 | |
| BP | GO:0002294 | | CD4-positive, alpha-beta T cell differentiation involved in immune response | | | 24/4223 | 68/18723 | | 0.0113655 | 0.14813191 | 0.13527971 | | 24 | |
| BP | GO:0032946 | | positive regulation of mononuclear cell proliferation | | | 43/4223 | 138/18723 | | 0.01198988 | 0.15120835 | 0.13808924 | | 43 | |
| BP | GO:0046632 | | alpha-beta T cell differentiation | | | 36/4223 | 112/18723 | | 0.01228941 | 0.15467273 | 0.14125304 | | 36 | |
| BP | GO:0032729 | | positive regulation of interferon-gamma production | | | 25/4223 | 72/18723 | | 0.01240126 | 0.15576578 | 0.14225125 | | 25 | |
| BP | GO:0042102 | | positive regulation of T cell proliferation | | | 33/4223 | 101/18723 | | 0.01243557 | 0.15588244 | 0.14235779 | | 33 | |
| BP | GO:0043367 | | CD4-positive, alpha-beta T cell differentiation | | | 28/4223 | 83/18723 | | 0.0129283 | 0.16173361 | 0.1477013 | | 28 | |
| BP | GO:0070661 | | leukocyte proliferation | | | 89/4223 | 318/18723 | | 0.0130063 | 0.16238324 | 0.14829457 | | 89 | |
| BP | GO:0002287 | | alpha-beta T cell activation involved in immune response | | | 24/4223 | 69/18723 | | 0.01378139 | 0.16777913 | 0.1532223 | | 24 | |
| BP | GO:0002293 | | alpha-beta T cell differentiation involved in immune response | | | 24/4223 | 69/18723 | | 0.01378139 | 0.16777913 | 0.1532223 | | 24 | |
| BP | GO:0070301 | | cellular response to hydrogen peroxide | | | 32/4223 | 98/18723 | | 0.01381634 | 0.16777913 | 0.1532223 | | 32 | |
| BP | GO:0072539 | | T-helper 17 cell differentiation | | | 12/4223 | 29/18723 | | 0.01808223 | 0.19868134 | 0.18144338 | | 12 | |
| BP | GO:0002292 | | T cell differentiation involved in immune response | | | 25/4223 | 75/18723 | | 0.021128 | 0.21865018 | 0.19967968 | | 25 | |
| BP | GO:0071236 | | cellular response to antibiotic | | | 7/4223 | 14/18723 | | 0.02230589 | 0.22011632 | 0.20101862 | | 7 | |
| BP | GO:0035710 | | CD4-positive, alpha-beta T cell activation | | | 32/4223 | 102/18723 | | 0.02481589 | 0.23748536 | 0.21688069 | | 32 | |
| BP | GO:0045582 | | positive regulation of T cell differentiation | | | 29/4223 | 91/18723 | | 0.02566384 | 0.2441003 | 0.22292171 | | 29 | |
| BP | GO:0002697 | | regulation of immune effector process | | | 92/4223 | 339/18723 | | 0.02606325 | 0.24676824 | 0.22535817 | | 92 | |
| BP | GO:1901990 | | regulation of mitotic cell cycle phase transition | | | 82/4223 | 299/18723 | | 0.02681641 | 0.25047408 | 0.22874249 | | 82 | |
| BP | GO:1902107 | | positive regulation of leukocyte differentiation | | | 46/4223 | 157/18723 | | 0.02912039 | 0.26818563 | 0.24491735 | | 46 | |
| BP | GO:1903708 | | positive regulation of hemopoiesis | | | 46/4223 | 157/18723 | | 0.02912039 | 0.26818563 | 0.24491735 | | 46 | |
| BP | GO:0070665 | | positive regulation of leukocyte proliferation | | | 44/4223 | 150/18723 | | 0.03166586 | 0.28385367 | 0.259226 | | 44 | |
| BP | GO:0046394 | | carboxylic acid biosynthetic process | | | 85/4223 | 314/18723 | | 0.0332034 | 0.29114729 | 0.26588681 | | 85 | |
| BP | GO:0001819 | | positive regulation of cytokine production | | | 122/4223 | 467/18723 | | 0.0365388 | 0.30392089 | 0.27755215 | | 122 | |
| BP | GO:0016053 | | organic acid biosynthetic process | | | 85/4223 | 316/18723 | | 0.03825488 | 0.31524853 | 0.28789699 | | 85 | |
| BP | GO:0034113 | | heterotypic cell-cell adhesion | | | 20/4223 | 61/18723 | | 0.04318926 | 0.34189212 | 0.31222892 | | 20 | |
| CC | GO:0101002 | | ficolin-1-rich granule | | | 55/4406 | 185/19550 | | 0.01361983 | 0.0646931 | 0.05049757 | | 55 | |

**Supplementary Table 8.** GO and KEGG analysis results of 57 common regulatory pathways in CD4+ T cells from AR and CSU patients.

**Supplementary Table 9.** Logistic regression model of 57 regulatory pathways and 6 pathways of DMG enrichment in 30 samples (For a full description of the statistics see additional Word file **Supplementary Table 9**).
