# Supplementary material for: DNA methylation regulatory patterns and underlying pathways behind the co-pathogenesis of allergic rhinitis and chronic spontaneous urticaria
Source: Front Immunol. 2023 Jan 11;13:1053558. doi: 10.3389/fimmu.2022.1053558 (PMC9875140; doi:10.3389/fimmu.2022.1053558)
Supplement: Supplementary file 1 [file DataSheet_1.zip › Supplementary Table 9.docx]

| **Description** | **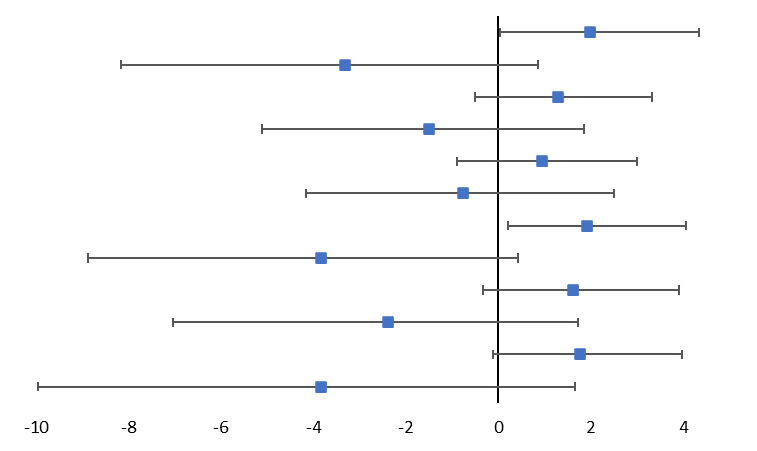lnOR（95%CI）** |  |  |  |  |  |  |  | **P value** |
| --- | --- | --- | --- | --- | --- | --- | --- | --- | --- |
| regulation of cell size | 1.987（0.034-4.340） |  |  |  |  |  |  |  | 0.064 |
|  | -3.310（-8.159-0.860） |  |  |  |  |  |  |  | 0.139 |
| developmental growth involved in morphogenesis | 1.293（-0.500-3.323） |  |  |  |  |  |  |  | 0.175 |
|  | -1.506（-5.116-1.859） |  |  |  |  |  |  |  | 0.384 |
| positive regulation of binding | 0.955（-0.898-3.005） |  |  |  |  |  |  |  | 0.326 |
|  | -0.764（-4.169-2.506） |  |  |  |  |  |  |  | 0.645 |
| regulation of cellular component size | 1.928（0.221-4.061） |  |  |  |  |  |  |  | 0.043 |
|  | -3.829（-8.871-0.428） |  |  |  |  |  |  |  | 0.098 |
| regulation of Ras protein signal transduction | 1.608（-0.337-3.906） |  |  |  |  |  |  |  | 0.127 |
|  | -2.389（-7.037-1.714） |  |  |  |  |  |  |  | 0.270 |
| axonogenesis | 1.770（-0.118-3.972） |  |  |  |  |  |  |  | 0.083 |
|  | -3.842（-9.949-1.661） |  |  |  |  |  |  |  | 0.185 |
|  |  |  |  |  |  |  |  |  |  |

**Supplementary Table 9**. Logistic regression model of 57 regulatory pathways and 6 pathways of DMG enrichment in 30 samples.
